# Alcohol-Related Thiamine Deficiency: Impact on Cognitive and Memory Functioning

**Published:** 1995

**Authors:** Philip J. Langlais

**Affiliations:** Philip J. Langlais, Ph.D., is a professor in the Department of Neurosciences at the University of California San Diego, School of Medicine, in the Neurology Service of the San Diego Veterans Affairs Medical Center, and in the Department of Psychology, San Diego State University, San Diego, California

**Keywords:** thiamine deficiency, cognitive process, AOD impairment, chronic AODE (alcohol and other drug effects), Wernicke-Korsakoff psychosis, brain

## Abstract

Chronic alcohol abuse is associated with several neurological disorders, including Wernicke-Korsakoff syndrome (WKS). Deficiency of thiamine—a vitamin essential for the metabolism and function of brain cells—is thought to be one factor contributing to the cognitive deficits and brain pathology characteristic of WKS. Excessive alcohol consumption may contribute to thiamine deficiency in several ways. Studies in human patients and animal models of WKS are attempting to identify brain structures affected by thiamine deficiency and to correlate them with the cognitive deficits observed in WKS patients.

Alcohol abuse can be associated with structural brain damage and disturbed cognition, memory, intellect, and personality. The clinical features of alcohol’s effects on the brain are heterogeneous and include mild to moderate cognitive impairment, amnesia, and dementia. The most commonly identified neurological disorder associated with chronic alcohol abuse is Wernicke-Korsakoff syndrome (WKS).^1^ Its most characteristic symptoms are anterograde amnesia, (i.e., the inability to learn and form new memories), retrograde amnesia (i.e., the loss of memories formed prior to the onset of WKS), and impairment of several cognitive processes. For more information on the cognitive deficits of patients with WKS, see sidebar, pp. 116–117.) These cognitive and memory deficits are accompanied by characteristic pathological damage to several brain structures.

**Cognitive Deficits in Alcoholic Wernicke-Korsakoff Syndrome**

Patients with Wernicke-Korsakoff syndrome (WKS) display several characteristic behavioral features. These include anterograde amnesia (i.e., the inability to form new memories), retrograde amnesia (i.e., the loss of memories formed prior to the onset of WKS), and impairment of several cognitive processes. Although the vast majority of patients develop WKS as a consequence of chronic alcohol abuse, these symptoms also apply to the non-alcoholic WKS patients. Retrograde amnesia is an interesting but poorly understood feature of WKS and will not be discussed here.

The most striking feature of WKS patients is their anterograde amnesia. For example, WKS patients may need weeks of practice to learn the route from their hospital room to the cafeteria. Similarly, within a few minutes following an extensive interview or testing session, WKS patients can fail to recall any details of the interview, the name of the interviewer, or even that the meeting took place. The cognitive nature and biological basis for these learning and memory deficits still are controversial. During the 1960’s and 1970’s, two schools of thought emerged. One school suggested that the impairment results from an inability to retrieve stored information from memory storage systems (Warrington and Weiskrantz 1970, 1974). The other school, which now is accepted more widely, places the deficit at the stage of information acquisition. According to this theory, new information is processed inadequately, resulting in incorrect encoding and storage of the information (Butters and Cermak 1980).

Several cognitive functions are disturbed in WKS patients and contribute to the learning and memory impairments. First, WKS patients have impaired attention (Talland 1965). The patients are highly susceptible to distraction, unable to screen out irrelevant information, fail to identify and focus on important features, and exhibit lapses in concentration. These behavioral symptoms are accompanied by anatomical changes in several structures in the brain stem and thalamus that control arousal and attention.

Second, WKS patients have perceptual impairments that may be related to their attention deficits. For example, compared with healthy people, WKS patients have more difficulty completing fragmented pictures or determining whether two odors, colors, or short musical passages are the same or different (Mair et al. 1986). These cognitive-perceptual capacities are mediated by neural systems located in the thalamus and in higher cortical regions that are damaged in WKS patients.

Third, WKS subjects demonstrate linguistic processing impairments reflected in alterations in the depth or degree to which they analyze words and sentences. For example, in a recent study by Cermak (1993), healthy people and WKS patients were asked to evaluate words on an orthographic level (i.e., whether the words were written in lower or upper case), on a phonemic level (i.e., whether the words rhymed), or on a semantic level (i.e., whether the words fit into a sentence). After the evaluation, the subjects had to recall the words. Healthy subjects’ performance was sensitive to the initial level of analysis: They recalled 15 percent of the words after orthographic analysis, 45 percent after phonemic analysis, and about 65 percent after semantic analysis. WKS subjects, in contrast, recalled words at similar rates (15 percent) irrespective of the level of initial evaluation. These observations suggest that WKS patients cognitively manipulate verbal information to a lesser degree than healthy people. Damage to thalamic-cortical systems in the left brain hemisphere of WKS patients may contribute to these linguistic processing deficits.

Fourth, WKS patients frequently are diagnosed with dementia, or global intellectual impairment. This includes loss of verbal and mathematical skills, inability to think abstractly, and a breakdown in social and personal hygiene skills. WKS patients also display personality changes characterized by general disinterest in ongoing events; little or no spontaneous emotion; and reduced reactions to unusual, frightening, or sexual stimuli (Butters and Cermak 1980; Victor et al. 1989). The incidence of dementia among WKS patients has not been studied thoroughly, but one study found significant intellectual deterioration in 60 percent of WKS cases (Squire and Shimamura 1986). Also, many patients diagnosed with WKS through post mortem brain studies previously had been diagnosed with dementia (Harper et al. 1986; Torvik et al. 1982).

One currently held view is that two factors contribute to the cognitive impairment and anatomical damage found in alcoholic WKS patients (figure 1). According to this view, alcohol-induced thiamine deficiency is responsible for the anterograde amnesia and damage to the diencephalon (i.e., the thalamus and the mammillary bodies). In addition, direct toxic effects of alcohol lead to the observed global intellectual decline and contribute to cortical damage, particularly in the frontal lobes, and to cortical shrinkage (Jacobsen and Lishman 1990). This hypothesis is supported by observations of severe cognitive and intellectual deficits indicative of dementia in alcoholics who do not exhibit the clinical symptoms of thiamine deficiency-induced Wernicke’s encephalopathy (e.g., ataxia and confusion). This phenomenon has been called alcoholic dementia. However, some recent studies suggest that thiamine deficiency also may be at least partly responsible for the cortical damage of WKS (for more information, see main article).

**Figure 1:**
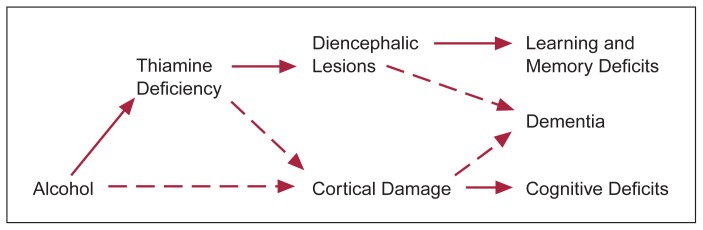
The proposed contribution of alcohol and thiamine deficiency to alcohol-related neuroanatomical and psychological dysfunction. Interrupted arrows indicate relationships that have not been established unequivocally.

The evolution of cognitive, behavioral, and memory impairments varies among WKS patients and may depend on the severity and frequency of thiamine deficiency. In some patients, amnesia and dementia develop gradually with slowly increasing severity; other patients develop symptoms in the absence of an acute episode of thiamine deficiency (i.e., without acute Wernicke’s encephalopathy). Furthermore, cognitive and memory impairments can improve to various degrees in many patients with time (Victor et al. 1989). The physiological basis for the recovery of mental functions is unknown, but studies of WKS subjects and an animal model of WKS suggest that undamaged cognitive brain systems can compensate for the lost cognitive functions (Cermak 1993; Savage and Langlais 1995).

— *Philip J. Langlais*

ReferencesButtersNCermakLSAlcoholic Korsakoff’s Syndrome: An Information Processing Approach to AmnesiaNew YorkAcademic Press19801011CermakLSMemory deficits in alcoholic Korsakoff patientsHuntWANixonSJAlcohol-Induced Brain DamageNational Institute on Alcohol Abuse and Alcoholism Research Monograph No. 22NIH Pub. No. 93–3545Bethesda, MDthe Institute1993157171HarperCGGilesMFinlay-JonesRClinical signs in the Wernicke-Korsakoff complex: A retrospective analysis of 131 cases diagnosed at autopsyJournal of Neurology, Neurosurgery, and Psychiatry49341345198610.1136/jnnp.49.4.341PMC10287563701343JacobsenRRLishmanWACortical and diencephalic lesions in Korsakoff’s syndrome: A clinical and CT scan studyPsychological Medicine2063751990232069910.1017/s0033291700013234MairRGDotyRKellyKMWilsonCLanglaisPJMc EnteeWJVollmeckeTAMultimodal discrimination deficits in Korsakoff amnesicsNeuropsychologia248318391986243364010.1016/0028-3932(86)90082-5SavageLMLanglaisPJDifferential outcomes attenuate memory impairments on matching-to-position following pyrithiamine-induced thiamine deficiency in ratsPsychobiology231531601995SquireLRShimamuraAPCharacterizing amnesic patients for neurobehavioral studyBehavioral Neuroscience1008668771986381434110.1037//0735-7044.100.6.866TallandGADeranged Memory: A Psychonomic Study of the Amnesic SyndromeNew YorkAcademic Press1965TorvikALindboeCFRogdeSBrain lesions in alcoholics: A neuropathological study with clinical correlationsJournal of the Neurological Sciences562332481982717554910.1016/0022-510x(82)90145-9VictorMAdamsRDCollinsGHThe Wernicke-Korsakoff Syndrome and Related Neurological Disorders due to Alcoholism and MalnutritionPhiladelphiaF.A. Davis1989WarringtonEKWeiskrantzLAmnesic syndrome: Consolidation or retrieval?Nature2286286301970499085310.1038/228628a0WarringtonEKWeiskrantzLThe effect of prior learning on subsequent retention in amnesic patientsNeuropsychologia124194281974443774010.1016/0028-3932(74)90072-4

WKS is a relatively frequent complication of alcohol abuse and, consequently, is not uncommon in the general population. Among 8,735 post mortem analyses conducted by Torvik and colleagues (1982), brain damage characteristic of WKS was detected in 12.5 percent of all alcoholics and in 0.8 percent of all brains examined. These data are consistent with an Australian study that found brain pathology indicative of WKS in 2.1 percent of a sample from the general population (Harper et al. 1989).

The diagnosis of WKS combines the acute neurological symptoms of Wernicke’s encephalopathy and the chronic amnesia of the Korsakoff state. Wernicke’s encephalopathy is an acute disorder, characterized by confusion, incoordinated gait, and abnormal eye movements. Anatomically, the brains of patients with Wernicke’s encephalopathy consistently show lesions in the diencephalon (i.e., the brain region encompassing the thalamus and the mammillary bodies), the cerebellum, and the brain stem (for the location of these and other brain structures, see figure 1 as well as the central brain figure and glossary on pp. 136–137). Other structures, such as the hippocampus and basal fore-brain, also may be damaged (Mayes et al. 1988; Jernigan et al. 1991). The disorder is thought to be caused primarily by a deficiency of thiamine (vitamin B) in the body, and a wide variety of mammalian species, including man, can develop it (Victor et al. 1989; Witt 1985). In developed countries, thiamine deficiency most commonly is a consequence of alcoholism, as described below. However, other conditions also can cause thiamine deficiency and Wernicke’s encephalopathy.

The acute neurological features of Wernicke’s encephalopathy often can be partially or completely reversed by thiamine administration. After treatment, however, approximately 50 to 65 percent of patients with Wernicke’s encephalopathy retain “an abnormality of mentation, in which learning and memory are affected out of proportion to other cognitive functions in an otherwise alert and responsive patient” (Victor et al. 1989, p. 11). This chronic amnesic stage is known as Korsakoff’s state. With prolonged thiamine treatment and abstention from alcohol, the amnesia also can improve in many patients. Victor and colleagues (1989) found that among their patients, almost all of whom were alcoholic, equal proportions either fully recovered their memory functions within 3 to 5 years, experienced significant but incomplete recovery, experienced slight recovery, or showed no measurable improvement of learning and memory functions.

This article reviews the roles of alcohol and thiamine deficiency in WKS development. Animal models of WKS suggesting that thiamine deficiency alone may be sufficient to produce learning and pathological changes similar to those observed in WKS also will be discussed. These findings suggest that it may not be alcohol’s neurotoxicity but rather thiamine deficiency—which may be caused or augmented by chronic alcohol consumption—that may be responsible for the cognitive deficits and anatomic changes seen in WKS patients.

## The Role of Thiamine Deficiency in the Development of WKS

Alcoholics often have several risk factors that may contribute to cognitive deficits and brain damage. These risk factors include fetal alcohol syndrome, drinking history, nutritional status, medical problems, polysubstance abuse, liver disease, head trauma, seizures, and cognitive state prior to drinking. The multiplicity of factors makes it difficult to assess the contribution of thiamine deficiency to cognitive impairment and WKS. Support for thiamine deficiency’s role comes from cases of nonalcoholic patients who develop WKS following acute nutritional deficiency (e.g., after chronic gastritis, stomach cancer, and constant vomiting) (for review, see Witt 1985). In one autopsy study, for example, 23 percent of cases with Wernicke’s encephalopathy were nonalcoholic (Lindboe and Loberg 1989). Thiamine’s normal role in brain functioning, as well as evidence from studies of human alcoholics and animal models of WKS, further suggests that thiamine deficiency may be one of the critical factors in the development of the amnesia and dementia associated with chronic alcohol abuse.

### Thiamine’s Role in Brain Functioning

Brain cells require thiamine to maintain adequate functions of several enzymes that are essential to the cells’ metabolism and functioning. In addition, thiamine itself also may play a vital role in maintaining cell membrane structure, excitability of the nerve cells, and the conduction of nerve impulses (Cooper and Pincus 1979; Iwata 1982). Because brain cells cannot synthesize thiamine, the vitamin must be absorbed from the diet through the intestinal tract and transported to the brain.

**Figure 2:**
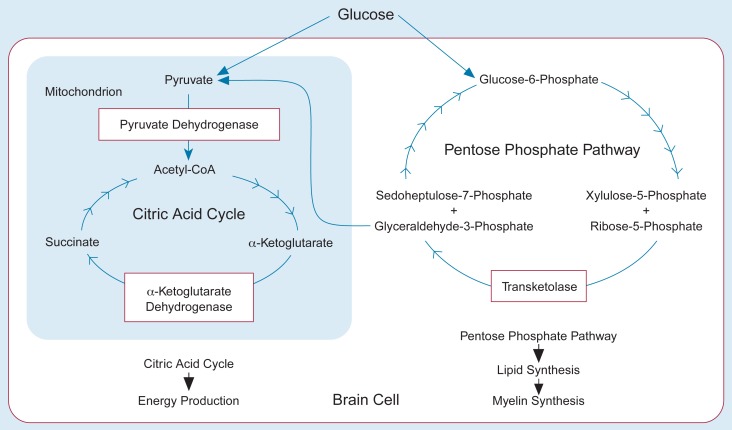
The role of thiamine-dependent enzymes in brain cell glucose metabolism. Glucose is the starting material, or substrate, for the pentose phosphate pathway and the citric acid cycle. The pentose phosphate pathway is a series of chemical reactions involved in the synthesis of lipids and of myelin (i.e., the substance comprising the sheaths around nerve cell axons). An intermediate product of the pentose phosphate pathway, glyceraldehyde-3-phosphate, is also the substrate for the citric acid cycle, which generates most of the energy needed to maintain cellular functions. The citric acid cycle takes place in the mitochondria, small organelles that also are called the cells’ “energy factories.” The figure shows some of the intermediate substances in the two pathways that are substrates and products of the reactions mediated by the thiamine-dependent enzymes transketolase, pyruvate dehydrogenase, and alpha-ketoglutarate dehydrogenase.

Three important enzyme systems involved in brain glucose metabolism require thiamine (figure 2). Two of these enzyme systems, the alpha-ketoglutarate dehydrogenase and the pyruvate dehydrogenase complexes, play key roles in the brain cells’ energy production. For example, a severe and prolonged decrease in alpha-ketoglutarate activity compromises cellular energy production and eventually can cause cell death.

The third thiamine-dependent enzyme, transketolase, is a key component of an energy-producing pathway that is involved in lipid synthesis. This pathway is essential to the maintenance and synthesis of myelin, the protective sheath surrounding axons that gives nerve fiber tracts their white appearance. Myelin is essential for proper conduction of nerve impulses from the nerve cell’s body to its distant terminals. In monkeys (Rinehart et al. 1949) and rodents (Watanabe and Kanabe 1978), one or more episodes of thiamine deficiency can cause the physical alteration and breakdown of brain myelin, presumably because of reduced transketolase activity. Depending on the brain region affected, this can lead to symptoms such as motor impairment (e.g., incoordinated gait or lack of balance), which also are found in WKS.

### Alcohol and Thiamine Deficiency

Normal thiamine uptake by the brain appears to just slightly exceed the amount required for proper brain functioning. Consequently, any reduction in thiamine availability could have serious effects on brain metabolism and may result in brain lesions and cognitive dysfunction. In alcoholics, several factors may contribute to thiamine deficiency.

First, nutritional thiamine deficiency can occur in alcoholics because of their poor eating habits. Alcoholics may eat nothing for days, and when they do eat, their food often is high in carbohydrates and low in vitamins such as thiamine. In addition, high carbohydrate intake further depletes the already low thiamine levels, because two enzymes involved in the breakdown of carbohydrates (i.e., pyruvate dehydrogenase and alpha-ketoglutarate dehydrogenase) are thiamine-requiring enzymes.

Second, alcoholics may develop a thiamine deficit because of impaired thiamine absorption from the intestine (Hoyumpa 1980). Alcohol damages the lining of the intestine and directly inhibits the transport mechanism that is responsible for thiamine absorption in the intestinal tract (Gastaldi et al. 1989). Also contributing to reduced thiamine uptake may be a nutritional deficiency of the vitamin folic acid, which commonly occurs in alcoholics as a consequence of poor nutrition. Studies in rats found that folic acid-deficient animals absorbed thiamine less efficiently than did healthy rats and that restoration of folic acid intake reversed the thiamine malabsorption (Howard et al. 1974).

Third, during chronic alcohol exposure, the activity of thiamine-metabolizing enzymes in the brain is compromised. To serve as a cofactor for the enzymes involved in energy production and lipid synthesis, thiamine must be converted into an active form by the enzyme thiamine pyrophosphokinase. Excessive alcohol consumption results in a significant decrease in thiamine pyrophosphokinase activity (LaForenza et al. 1990). In addition, alcohol consumption increases the activity of the enzymes that break down activated thiamine in the brain (LaForenza et al. 1990). Through these mechanisms, alcohol could reduce the activity of thiamine-dependent enzymes and affect brain metabolism even in the presence of adequate nutrition and thiamine absorption.

## Thiamine Deficiency, Diencephalic Lesions, and Learning and Memory Deficits

Studies in human WKS patients and in animal models of WKS have shown characteristic damage to the diencephalon, including loss of nerve cells in the thalamus, cell death and hemorrhages in the mammillary bodies, and enlargement of the third ventricle. The results of studies in humans and animals seeking to establish the links between thiamine deficiency, diencephalic lesions, and the cognitive deficits observed in WKS are described below.

Damage to the hippocampus and the surrounding mesial temporal lobe—limbic structures involved in learning and memory—also has been reported in magnetic resonance imaging (MRI) studies of alcoholics with and without Korsakoff’s syndrome (Jernigan et al. 1991; Squire et al. 1990; Sullivan et al. 1995. The contribution of these changes to the learning and memory impairments is uncertain because the volume loss in these structures is relatively small (8–10 percent) and has not demonstrated a strong relationship to the subjects’ performance on memory tests (Squire et al. 1990). Post mortem anatomical studies of alcoholic patients also provide little information about the status of these structures (Harper and Kril 1993), despite the extensive literature describing hippocampal damage in experimental models of alcohol toxicity (for a review, see Walker et al. 1993). Routine anatomical examination of the brains of animals recovered from acute thiamine deficiency have failed to detect any gross abnormalities of hippocampal or related mesial temporal lobe structures (Langlais 1992; Langlais et al. 1992). However, the hippocampus is a long, complex structure with densely packed neurons that make detection of even moderate cell loss difficult. More careful studies are needed to determine the effects, if any, of thiamine deficiency on the hippocampus and related temporal lobe structures.

### Human Studies

Both autopsy studies and imaging technologies, such as computer tomography and MRI, can detect the anatomical damage characteristic of Wernicke’s encephalopathy in alcoholic and nonalcoholic WKS patients who have a wide range of cognitive and memory impairments (Harper and Kril 1993; Pfefferbaum and Rosenbloom 1993). Some of these diencephalic lesions also are detectable in chronic alcoholics who, based on their behavioral and cognitive functioning, are not diagnosed with WKS (Pfefferbaum and Rosenbloom 1993). This finding suggests that in some cases (e.g., after mild bouts of thiamine deficiency) diencephalic lesions may develop without the typical neurological features seen after severe thiamine deficiency.

Several hypotheses exist regarding the precise contribution of diencephalic lesions to the cognitive and the anterograde learning and memory deficits of WKS (Mayes et al. 1988). The most common hypothesis suggests that the variability and severity of the cognitive and memory deficits in WKS reflect the extent of damage to two major learning and memory systems located in the temporal lobe and the diencephalon. The first memory system includes nerve fibers connecting the hippocampus, mammillary body, anterior nucleus of the thalamus, and cingulate cortex. The second system comprises nerve fibers connecting the amygdala, mediodorsal nucleus of the thalamus, and frontal cortex. A recent theory attributes explicit memory (e.g., the conscious recollection of facts or events) to diencephalic and mesial temporal lobe structures, priming (i.e., unconscious memory) to the neocortex, and skill learning to the striatum (see Jernigan and Ostergaard, pp. 104–107).

Studies of amnesia following strokes in the thalamus demonstrate that the location and extent of damage to these various diencephalic pathways and structures are critical determinants of the type and severity of cognitive and memory dysfunctions observed (Cramon et al. 1985; Malamut et al. 1992). The contribution of damage to specific thalamic regions to memory impairment was demonstrated by an MRI study of amnesic alcoholic Korsakoff patients and nonamnesic alcoholics (Jernigan et al. 1991). Both groups displayed shrinkage of the diencephalon, especially of its posterior regions. However, only the amnesic alcoholic Korsakoff patients displayed significant shrinkage of the anterior thalamus.

Certain thalamic nuclei and fiber tracts often damaged in WKS affect not only memory systems but also the following cognitive functions, all of which are disrupted in alcoholic WKS patients:

- Nerve fibers from thalamic nuclei often damaged in WKS, including the mediodorsal and intralaminar nuclei, extend to areas of the prefrontal cortex that are important for planning, organizing, and selecting behavioral responses (Markowitsch 1982).
- Nerve fibers extending from the anterior nucleus of the thalamus to the cingulate cortex play an important role in discriminative learning (Gabriel 1993) and frequently are damaged in human WKS.
- The intralaminar system, which consists of the intralaminar nuclei of the thalamus and nerve fibers connecting them to the cortex and which can be affected in WKS, is important for behavioral arousal and plays a critical role in regulating activity of cortical neurons (Steriade and Glenn 1982). Disruption of the intralaminar system may be associated with the attention deficit and impaired information processing observed in some WKS patients and may contribute to their learning impairment.
- Thalamic sites often damaged in WKS also include fibers connecting the cerebellum and basal ganglia with the thalamus (Haroian et al. 1981; Gerfen et al. 1982). These complex circuits may be involved in evaluating competing sensory-memory information and in preparing and selecting motor responses (Johnstone and Rolls 1990).

This anatomic evidence suggests that lesions in the diencephalon also may be associated with cognitive impairments ascribed to frontal lobe damage and to dementia induced by extensive cortical damage. This hypothesis is supported by findings among 70 alcoholics that dementia was associated with thalamic and mammillary body lesions but was not always predictive of cortical atrophy (Torvik et al. 1982). Harper and colleagues (1989) drew similar conclusions in their study of 131 alcoholics with diencephalic lesions. These researchers hypothesize that most alcoholics with dementia at autopsy will demonstrate the lesions characteristic of thiamine deficiency-induced Wernicke’s encephalopathy.

### Animal Studies

Animal studies have contributed significantly to our understanding of the role of thiamine deficiency in the anatomical and behavioral changes associated with chronic alcohol abuse. However, there are formidable structural, anatomical, and behavioral differences between experimental animals and humans that limit the interpretation of data obtained from these studies.

Animal models have used two different approaches to induce thiamine deficiency. The older method restricts dietary thiamine intake by feeding a diet that contains little or no thiamine. Dietary restriction can be applied for one or more periods, typically lasting 3 to 4 weeks. This method has been used with varying degrees of success in monkeys and rats to study the resulting anatomical and neurological symptoms.

One study using a single period of thiamine restriction in monkeys failed to produce the diencephalic damage characteristic of WKS. Instead, lesions were limited to the brain stem and cerebellum (Blank et al. 1975). In another study, Rinehart and colleagues (1949) subjected rhesus monkeys to an unspecified number of episodes of thiamine deficiency. The animals displayed lesions in the thalamus, mammillary body, and basal ganglia as well as in the brain stem and cerebellum. In another study, monkeys underwent multiple periods of thiamine deprivation. These animals showed many of the neurological signs observed in humans with WKS, including anorexia, incoordinated gait, abnormal eye movement, and confusion (Mesulam et al. 1977). However, none of these earlier studies in monkeys examined learning and memory capacities following recovery from thiamine deficiency. Subsequent studies have concluded that in monkeys, two or more periods of thiamine deficiency are required for the animals to develop thalamic and mammillary body lesions as well as the learning and memory deficits typical of human WKS (Witt and Goldman-Rakic 1983*a*,*b*).

The second and more recent method of producing thiamine deficiency in animal models uses a thiamine antagonist either alone or in combination with dietary restriction. The most commonly used thiamine antagonist, pyrithiamine, inhibits the conversion of thiamine to its active form in the brain. Several studies have shown that pyrithiamine-induced thiamine deficiency (PTD) in rats leads to neurologic symptoms similar to that observed in thiamine-deficient monkeys and humans with WKS (Langlais 1992; Langlais and Savage 1995; Troncoso et al. 1981). More importantly, PTD elicits a pattern of widespread brain damage in the rat—especially in the diencephalon—that is remarkably similar to the pathological changes in human WKS (Troncoso et al. 1981). And several weeks after a single acute bout of thiamine deficiency, PTD rats demonstrated impaired learning and memory capacities on a wide variety of tasks (Langlais 1992; Langlais et al. 1992; Langlais and Savage 1995; Mair et al. 1991*a*,*b*). These impairments correlated strongly with damage to medial and intralaminar regions of the thalamus and the mammillary body. Some of the rats recovering from PTD also exhibited wide-ranging cognitive impairment, accompanied by significant shrinkage of the corpus callosum (i.e., the main myelinated fiber tract connecting the two cerebral hemispheres) and the frontal and parietal cortices (Langlais and Savage 1995; Savage and Langlais 1995). The importance of these latter observations to the role of thiamine deficiency in cortical damage and cognitive deficits will be discussed below.

Several cognitive disturbances may account for the learning and memory impairments observed after thiamine deficiency in the rat. A cognitive system located in the mediodorsal nucleus of the thalamus and the frontal cortex—which is considered essential for evaluating probable outcomes, the temporal sequence of ongoing and previous events, the initiation of appropriate responses, and other higher cognitive functions—frequently is structurally damaged after thiamine deficiency. Alternatively, impaired attention due to damage to the brain stem and the thalamus may contribute to the learning deficits by heightening distractibility and thus preventing proper information processing and discrimination of relevant from irrelevant stimuli.

For example, on the standard delayed response task, rats must remember in which of two goal boxes they received food on the previous trial. Recovered PTD rats are significantly impaired on this task. However, when the task is modified to provide more specific types of reinforcement and thus more cues to distinguish among response choices, the PTD rats perform as well as normal rats (Savage and Langlais 1995), indicating that with additional cues, performance can return to control levels. Analogously, WKS patients perform as well as controls if they are aided by procedures that enhance their attention to and processing of details of the task (Cermak 1993). The observations in recovered PTD rats support Cermak’s theory that WKS patients can compensate for cognitive deficits with alternative mechanisms when learning new tasks.

When rats are subjected to identical bouts of thiamine deficiency, the animals show significant individual variations in the extent of brain damage and the degree of learning and memory impairment (Langlais et al. 1992; Langlais and Savage 1995; Mair et al. 1991*a*,*b*; Savage and Langlais 1995). Some animals fail to demonstrate any significant damage to the thalamus or the mammillary body or any behavioral impairment. Other animals display anatomical changes similar to those observed in Wernicke’s encephalopathy and varying degrees of impairments—from mild deficits to the total inability to learn new tasks. These individual variations in brain lesions and behavioral impairments are reminiscent of the variability of symptoms among human WKS patients. Genetic differences among people may play a significant role in determining differential responses to an equivalent thiamine deprivation. Using the PTD method in rodents with known genetic backgrounds may be a useful approach for future investigations of the genetic factors underlying susceptibility to thiamine deficiency.

## Thiamine Deficiency, Cortical Lesions, and Cognitive Deficits

### Human Studies

The cognitive deficits in WKS patients usually are accompanied by damage in the diencephalon and the cortex, which are connected through a multitude of nerve tracts. Some researchers have suggested that only the diencephalic lesions are due to thiamine deficiency, whereas cortical damage is caused by a direct toxic action of alcohol (Freund and Ballinger 1988). This theory is supported by findings that global shrinkage of the cortex, which is associated with cognitive and intellectual deficits, is present in alcoholics with and without diencephalic damage indicative of thiamine deficiency (Harper and Kril 1993).

However, although chronic alcohol consumption may lead to cortical damage, patients with persistent and severe cognitive deficits most commonly exhibit diencephalic lesions. Because the diencephalon and the cortex are highly interconnected through nerve fibers, damage to one of these brain regions (i.e., the diencephalon) can interfere with the functions of the other region (i.e., the cortex) and even may induce pathological damage. For example, thiamine deficiency-induced damage to the thalamic mediodorsal nucleus and subsequent loss of nerve fibers projecting from the nucleus to the frontal lobes of the cortex could contribute to the frontal lobe damage that frequently is observed in alcoholics (Jernigan et al. 1991; Harper and Kril 1993; Risberg and Berglund 1987).

### Animal Studies

Animal studies support the hypothesis that thiamine deficiency alone, in the absence of alcohol intake, can cause lesions in the cortex and in the myelinated fibers (white matter), in addition to the diencephalic damage. White matter damage and loss of fiber tracts have been observed in several mammalian species following thiamine deficiency (reviewed in Witt 1985). Recently, researchers have found significant reductions in the thickness of the corpus callosum and the frontal and parietal cortices of recovered PTD rats, deficits that are associated with severe behavioral impairments (Langlais and Savage 1995; Savage and Langlais 1995). These observations demonstrate that thiamine deficiency alone can produce measurable loss of both white matter tracts and cortical tissue.

PTD animals with more extensive damage to cortical and diencephalic structures displayed severe learning and memory deficits (Langlais and Savage 1995). They appeared to have difficulty integrating, associating, and retaining information about important features of a task; an inability to adapt response strategies; and heightened susceptibility to distraction. Perseveration (i.e., continuing to elicit the same behavioral response despite the absence of reinforcement) and an inability to shift strategies are indicative of frontal lobe dysfunction and are common features of human WKS.

Although the rats in these studies were subjected to a single acute bout of thiamine deficiency, resulting in diencephalic as well as cortical damage, a more recent study detected degenerating white matter fibers in the cortex even after a mild episode of thiamine deficiency (Langlais and Zhang in press). These animals exhibited little or no diencephalic damage, suggesting that a mild bout of thiamine deficiency can cause selective loss of cortical white matter without significantly damaging the mammillary body or thalamus. It seems possible, therefore, that repeated bouts of mild thiamine deficiency may produce a cumulative and significant loss of cortical white matter and nerve cells, leading to cognitive impairments in the absence of the classic diencephalic lesions of Wernicke’s encephalopathy.

## Conclusions

Several lines of evidence, from both human and experimental animal studies, suggest that thiamine deficiency is associated with the brain lesions and cognitive deficits observed in WKS. However, the molecular mechanisms causing the anatomical changes are unknown. Recent studies suggest the possible involvement of an excitotoxic mechanism (Langlais 1995). These studies found that during thiamine-deficient states, excess amounts of excitatory neurotransmitters are released into certain brain regions. In combination with reduced energy production associated with thiamine deficiency, the excess neurotransmitters lead to the destruction of nerve cells. Accordingly, treatment with drugs that block excitotoxicity has been shown to prevent both pathologic damage to the diencephalon and behavioral impairments (Langlais and Mair 1990; Robinson and Mair 1992).

The importance of thiamine deficiency in WKS does not exclude the possibility that direct alcohol neurotoxicity also contributes to the disorder, but the precise contributions of thiamine and alcohol remain to be determined. It will be difficult to address mechanistic questions in human studies because multiple medical, psychological, and neurological factors may affect cognitive functioning in alcoholic patients. Therefore, animal studies may be more suitable for examining the relative contributions of thiamine deficiency and alcohol neurotoxicity to cognitive impairments and to diencephalic and cortical brain damage.

Of particular interest is the observation that repeated mild bouts of thiamine deficiency produce more severe cognitive deficits and pathologic damage than a single acute bout. Consequently, future studies should pay particular attention to the nutritional status of alcoholics, especially their patterns of binge drinking and the concomitant fluctuations in nutrition and vitamin intake.

## Figures and Tables

**Figure 1:**
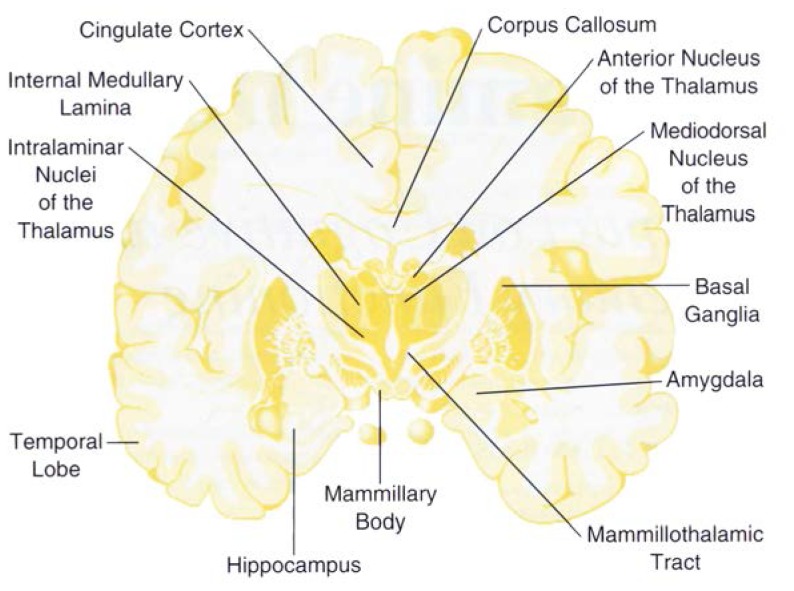
Schematic of a cross-section through the brain showing brain structures that are damaged in patients with Wernicke-Korsakoff syndrome. SOURCE: Adapted from Nieuwenhuys, R.; Voogd, J.; and van Huijzen, C. *The Human Central Nervous System: A Synopsis and Atlas*. New York: Springer Verlag, 1988.

## Footnotes

1 WKS is not limited to alcoholic patients; however, although no accurate studies have been published, it has been estimated that 80 to 90 percent of WKS patients are long-term alcohol abusers.
